# Transcriptomic Analysis on Pectoral Muscle of European Meat Pigeons and Shiqi Pigeons during Embryonic Development

**DOI:** 10.3390/ani13203267

**Published:** 2023-10-19

**Authors:** Fada Li, Chenyu Zhu, Yongquan Luo, Songchao Li, Qi Wang, Yuanhao Han, Zhongping Wu, Xiujin Li, Yayan Liang, Yitian Chen, Xu Shen, Yunmao Huang, Yunbo Tian, Xumeng Zhang

**Affiliations:** 1 Guangdong Laboratory for Lingnan Modern Agriculture, Guangzhou 510225, China; lifada@zhku.edu.cn (F.L.); zhuchenyu@zhku.edu.cn (C.Z.); lyq19991112@163.com (Y.L.); lsc_1128@163.com (S.L.); 15915541752@163.com (Q.W.); how9700@foxmail.com (Y.H.); wuzhongping@zhku.edu.cn (Z.W.); lixiujin@zhku.edu.cn (X.L.); sandy130130@163.com (Y.L.); chenyt1006@126.com (Y.C.); shenxu@zhku.edu.cn (X.S.); huangyunmao@zhku.edu.cn (Y.H.); 2 College of Animal Science and Technology, Zhongkai University of Agriculture and Engineering, Guangzhou 510225, China

**Keywords:** pigeon, pectoral muscle, myogenesis, transcriptome

## Abstract

**Simple Summary:**

The meat production performance of pigeons is largely determined by the growth and development of skeletal muscle, and the pectoral muscle accounts for a large proportion of skeletal muscle. The incubation period of pigeons is around 18 days. In order to better investigate the developmental phenotypic differences and hub differentially expressed genes (DEGs) in the pectoral muscles between the European meat pigeon Mimas strain and the Shiqi pigeon during the whole embryonic stages, we selected embryonic day 6 (E6), day 10 (E10), day 14 (E14) and day 1 after birth (P1), representing early embryonic development, mid-embryonic development, late-embryonic development, and the first day after hatching. H&E staining and RNA-seq at different embryonic stages were analyzed. It was found that myofiber density was significantly higher in the Shiqi pigeon than that of the European meat pigeon on E6, and myofibers with a diameter in the range of 1~50 μm in the Shiqi pigeon were significantly higher than those in the European meat pigeon on P1. In addition, we found several myogenic DEGs (*CLU*, *PTGS1*, *NXK6*-1, *NR1H4*, *HNF1A*, *ABCB11*, *PLA2G12B* and *BPHL*) which may play roles in regulating distinct embryonic pectoral muscle development between two pigeon breeds. This study provides basic data for revealing the distinct embryonic developmental mechanisms of pectoral muscle between European meat pigeons and Shiqi pigeons.

**Abstract:**

In avian muscle development, embryonic muscle development determines the number of myofibers after birth. Therefore, in this study, we investigated the phenotypic differences and the molecular mechanism of pectoral muscle development of the European meat pigeon Mimas strain (later called European meat pigeon) and Shiqi pigeon on embryonic day 6 (E6), day 10 (E10), day 14 (E14) and day 1 after birth (P1). The results showed that the myofiber density of the Shiqi pigeon was significantly higher than that of the European meat pigeon on E6, and myofibers with a diameter in the range of 50~100 μm of the Shiqi pigeon on P1 were significantly higher than those of European meat pigeon. A total of 204 differential expressed genes (DEGs) were obtained from RNA-seq analysis in comparison between pigeon breeds at the same stage. DEGs related to muscle development were found to significantly enrich the cellular amino acid catabolism, carboxylic acid catabolism, extracellular matrix receptor interaction, REDOX enzyme activity, calcium signaling pathway, ECM receptor interaction, PPAR signaling pathway and other pathways. Using Cytoscape software to create mutual mapping, we identified 33 candidate genes. RT-qPCR was performed to verify the 8 DEGs selected—*DES*, *MYOD*, *MYF6*, *PTGS1*, *MYF5*, *MYH1, MSTN* and *PPARG*—and the results were consistent with RNA-seq. This study provides basic data for revealing the distinct embryonic development mechanism of pectoral muscle between European meat pigeons and Shiqi pigeons.

## 1. Introduction

In recent years, the pigeon industry in China has been rapidly developed. The pigeon breeds raised in China are mainly the Shiqi pigeon, European meat pigeon, and Silver king pigeon. Shiqi pigeons have strong disease resistance and adaptability, rough feeding resistance, easy breeding, and good production performance, and the average weight of an adult pigeon is 700–800 g [1], and the average weight of 28-day-old squabs is 566.68 g in our unpublished investigation. Adult pigeons of the European meat pigeon Mimas strain weigh about 700~750 g [2], and the average weight of 28-day-old squabs is 601 g [3].

Pigeon meat has rich nutrition, high protein content and medicinal value and low-fat content. In China, pigeon meat is known as “animal ginseng” and is considered an advanced nutritional product, which is increasingly favored by consumers [4]. The growth and development of skeletal muscle determines its meat production performance [5], and pectoral muscle accounts for about 20% to 30% of its body weight [6]. Myogenesis is a complex process [7], during the embryonic stage, muscle progenitors undergo proliferation and differentiation processes to form myoblasts, which then fuse to form multinucleated myotubes. Finally, myotubes mature into myofibers with contractile properties [8]. The number of myofibers is determined during embryonic stages and remains unchanged after birth [9]. The formation, determination and eventual differentiation of muscle cells are largely controlled by a network of four muscle regulatory factors (MRFs): myogenic factor 5 (*MYF5*), muscle-specific regulatory factor 4 (*MRF4*), myogenic myocyte-determining protein (*MYOD*) and myogenin (*MYOG*) [10]. However, the regulatory network of skeletal muscle is complex, and many regulatory mechanisms and factors involved in pigeon embryonic pectoral muscle development have not been identified yet.

RNA-seq can explore differences in gene expression levels at a holistic level, directly linking them to phenotypic changes. Transcriptome expression has also been studied in some poultry pectoral and leg muscles at different embryonic and growth stages [11,12,13]. A previous study identified five genes related to skeletal muscle development and growth in Tarim pigeons at embryonic days 8 and 13, and postnatal days 1 and 10 by RNA-Seq analysis [14]. In another related study, RNA-seq characterized the expression profiles of lncRNA, miRNA and mRNA during skeletal muscle development in White King pigeons on embryonic days 8 and 13, and birth days 1 and 10 [15]. Four genes associated with muscle development were identified by RNA-seq in Shitou and Wuzong geese at embryonic days 15 and 30 and the first day of life [16]. Although several skeletal muscle transcriptomic studies concerning pigeons and other avian species have been reported, the distinct embryonic development mechanism of pectoral muscle between pigeon breeds differing in body size is still largely unknown.

In this study, we investigated the phenotypic differences and the molecular mechanism of pectoral muscle development of European meat pigeons and Shiqi pigeons throughout embryonic stages by using the RNA-seq method and laid the foundation for revealing the distinct embryonic developmental mechanisms of pectoral muscle between European meat pigeon and Shiqi pigeon.

## 2. Materials and Methods

### 2.1. Animal and Tissue Collection

Pectoral muscle samples of European meat pigeon and Shiqi pigeon were collected on embryonic day 6 (E6), day 10 (E10), day 14 (E14) and day 1 after birth (P1), three pigeons per breed per stage (*n* = 3) was collected, with a total of 24 pigeons. The right and left pectoral muscles of each pigeon were taken, with the left pectoral muscle being placed in paraformaldehyde for subsequent HE-stained sections and the right pectoral muscle being placed in liquid nitrogen and snap frozen at −80 °C in the refrigerator for subsequent experiments. The pigeons were obtained from Jinlv Modern Agriculture Development Co., Ltd., Meizhou, Guangdong, China.

### 2.2. Hematoxylin–Eosin (H&E) Staining of Pectoral Muscle

Pectoral muscles from three pigeons of each breed from each stage (*n* = 3) were utilized. Sections were processed by H&E staining. A 20.0× image of muscle tissue was captured for each section on CaseViewer 2.2 scanning and viewing software. One field of view was selected for each of the three biological replicates of each species in each period for analysis. The number and diameter (μm) of all myofiber in the view field of the section were counted by Adobe Photoshop 2021, and the density was calculated by the number and field area of the section. Cut the pectoral muscle still attached to the bone horizontally perpendicular to the myofiber, and draw the cross-sectional area on the transparent paper [17]. The total number of myofiber was calculated by muscle fiber density and cross-sectional area. Data were counted using each individual pigeon as a replicate. The histological statistic analysis data are shown in Supplementary Table S1.

### 2.3. RNA Extraction, Library Construction and Sequencing

Pectoral muscles from three pigeons of each breed from each stage (*n* = 3) were utilized. Total RNA was extracted using a Trizol kit (Invitrogen, Carlsbad, CA, USA) Manufacturer’s agreement. RNA quality was assessed on the Agilent 2100 bioanalyzer (Agilent Technologies, Palo Alto, CA, USA), and RNase-free agarose gel was used for detection electrophoresis. After total RNA extraction, eukaryotic mRNA was enriched with Oligo (dT). The enriched mRNA was then fragmented into short fragments using fragmentation buffer and reverse transcribed into cDNA using the NEBNext Ultra RNA Library Preparation Kit (NEB#7530, New England Biolabs, Ipswich, MA, USA). End repair of purified double-stranded cDNA fragments was performed. A base was added, and connected to the Illumina sequencing adapter. The ligation reaction was purified with AMPure XP Beads (1.0×). Ligated fragment size was selected by agarose gel electrophoresis and polymerase chain reaction (PCR) amplified. The cDNA library was sequenced by Illumina Novaseq6000 Gene Denovo Biotechnology Co. (Guangzhou, China).

### 2.4. Quality Control and Comparative Analysis

Reads obtained from the sequencer included raw reads that contained adapters or low-quality bases, in order to get high-quality clean reads, the reads need to be further filtered by fastp (version 0.18.0) [18]. The parameters were as follows: remove the reads containing the adapters; remove reads containing more than 10% unknown nucleotides (N); remove low-quality reads containing more than 50% of low-quality bases (Q value ≤ 20). Our sequencing was paired end with a read length of 150 bp, and the filtering statistics of sequencing data are shown in Supplementary Table S2. Reads were targeted to ribosomes using the short reads comparison tool Bowtie2 (version 2.2.8) RNA (rRNA) database [19]. The reading of the rRNA map would then be removed. The rest clean reads were further used for assembly and gene abundance calculation. An index of the reference genome was built, and paired-end clean reads were mapped to the reference genome using HISAT2. 2.4 with “-rna-strandness RF” and other parameters set as a default, and the reference genome is *Columba livia* (https://www.ncbi.nlm.nih.gov/assembly/GCA_000337935.2/, accessed on 13 December 2017) [20]. The average total reads in each sample was about 4.97 × 10^7^, and 79.6% reads were mapped to the genome on average (Supplementary Table S3). The number of reads involved in the quantification has been provided in the Supplementary Table S4.

### 2.5. Quantification of Gene Abundance

The mapped reads of each sample were assembled by using StringTie v1.3.1 in a reference-based approach [21,22]. For each transcribed region, FPKM (fragments per kilobase per million mapped reads transcripts) values were calculated to quantify their expression abundance and changes, using RSEM software v1.3.3 [23].

The FPKM formula is as follows:

$$FPKM=\frac{10^{6}C}{{NL/10}^{3}}$$

where FPKM—the expression amount of analyzed gene; C—the number of mapped fragments; N—the total number of fragments mapped to the internal reference gene; L—the length of analyzed gene [bases]. The FPKM method is able to eliminate the effects of different gene lengths and sequencing data volumes on gene expression calculations. Therefore, the calculated gene expression can be directly used to compare gene expression differences between samples.

### 2.6. Identification of Differentially Expressed Genes

DESeq2 software was used to analyze the differential expression genes (DEGs) between the two different groups [24]. Three samples in each comparison were used. The raw reads count was used for differential expression analysis by DESeq2. The analysis was divided into three main parts: (1) normalization of the read counts; (2) calculation of the probability of hypothesis testing (pvalue) according to the model; (3) finally, multiple hypothesis testing corrections are carried out to obtain the FDR value. The comparison performed were as follows: MME6-vs-SQE6, MME10-vs-SQE10, MME14-vs-SQE14, MMP1-vs-SQP1, SQE6-vs-SQE10, SQE10-SQE14, SQE14-vs-SQP1, MME6-vs-MME10, MME10-vs-MME14 and MME14-vs-MMP1. The genes/transcripts with the parameter of false discovery rate (FDR) below 0.05 and log2FC > 1 were considered differentially expressed genes/transcripts. According to the DEGs in each comparison group, volcano map analysis and hierarchical clustering were performed for the expression patterns of DEGs, and the clustering results were presented by heatmap.

### 2.7. Analysis of GO and KEGG Pathway Enrichment

The GO enrichment analysis provides all the GO terms that are significantly enriched by DEGs. It was compared with the genomic background and filtered out the DEGs corresponding to the organism features. First, map all DEGs to GO terms in the gene ontology database (http://www.geneontology.org/ (accessed on 23 November 2022)), then calculate gene numbers for the number of genes per term, and define GO terms that are significantly enriched in DEGs compared to the genomic background by hypergeometric tests.

The calculated *p*-value was corrected by FDR, with FDR ≤ 0.05 as a threshold. GO terms that satisfy this condition were defined as GO terms that were significantly enriched in DEGs. The analysis was able to identify the main biological functions of DEGs.

Pathway-based analysis can help to further understand the biological function of genes. KEGG is the main public access-related database [25]. Pathway significance enrichment analysis was performed in terms of the KEGG pathway and a hypergeometric test was applied to identify pathways that were significantly enriched in DESs compared to background genes. The calculated *p*-value was FDR corrected, taking FDR ≤ 0.05 as a threshold value.

### 2.8. Construct the Gene Interaction Network

We used STRING (https://cn.string-db.org/ (accessed on 13 January 2023)) to construct and screen gene interaction networks. This network contained genes with significant functional enrichment associated with muscle development. We only retained network edges that meet the confidence score >0.4. Visualize the gene–gene interaction on input using Cytoscape (v3.9.01), and the strength of gene interactions was reflected by the betweenness algorithm in this software (Supplementary Table S5).

### 2.9. Real-Time Quantitative PCR (RT-qPCR) Confirmation of DEGs

The same samples used in RNA-seq from three pigeons of each breed from each stage (*n* = 3) were utilized. The cDNA was synthesized by reverse transcription kit (GenStar, Beijing, China). RT-qPCR was performed in three batches using the SYBR Green qPCR kit (GenStar, Beijing, China) and tested in the LightCycler 480 II system (Roche, Basel, Switzerland). The expression of each gene was normalized to the *GAPDH* transcript. RT-qPCR data were processed by the 2^−∆∆CT^ method, and statistical analysis was then performed using Two-way ANOVA and Tukey-style multiple comparisons. The significance and correlation coefficient (r) analysis were calculated using GraphPad Prism 8.0. The primers used in this study are shown in Supplementary Table S6.

## 3. Results

### 3.1. Analysis of Pectoral Muscle H&E Staining Results of Two Pigeon Breeds

**Figure 1:**
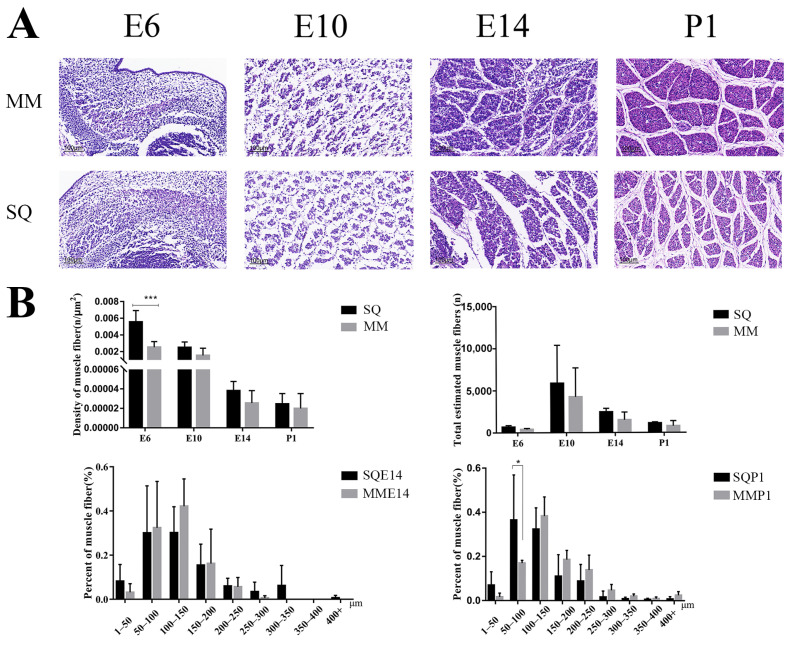
Phenotypic analysis of pectoral muscle in two pigeon breeds during embryonic stages. (**A**) H&E-stained sections of the pectoral muscles of two pigeon breeds during embryonic stages. Scale bar = 100 μm. (**B**) Myofiber density, estimated total myofiber number and myofiber diameter distribution ratio of two pigeon breeds during embryonic stages. SQ: Shiqi pigeon, MM: European meat pigeon, embryonic day 6 (E6), day 10 (E10), day 14 (E14) and day 1 after birth (P1), same below. *, *p* < 0.05; ***, *p* < 0.001.

To explore differences in myofiber development between the two pigeon breeds, we performed an H&E-stained section analysis. The results showed that the myofiber density and total number of myofibers in Shiqi pigeons were greater than those of European meat pigeons during all periods, and the myofiber density of Shiqi pigeons was significantly greater than that of European meat pigeons on E6 (Figure 1A,B). On E14, myofibers with a diameter in the range of 1~50 μm and 200 μm or more were greater in Shiqi pigeons than those in European meat pigeons, myofibers with diameters in other diameter ranges of European meat pigeons were greater than those of Shiqi pigeon but did not reach significance. On P1, myofibers with diameters in the range of 1~50 μm and 50~100 μm were greater in the Shiqi pigeon than those in the European meat pigeon, and myofibers with diameters in the other ranges were greater in the European meat pigeon than those in the Shiqi pigeon, and it reached significance in the 50~100 μm range (Figure 1B).

### 3.2. Overview of RNA-Seq

**Figure 2:**
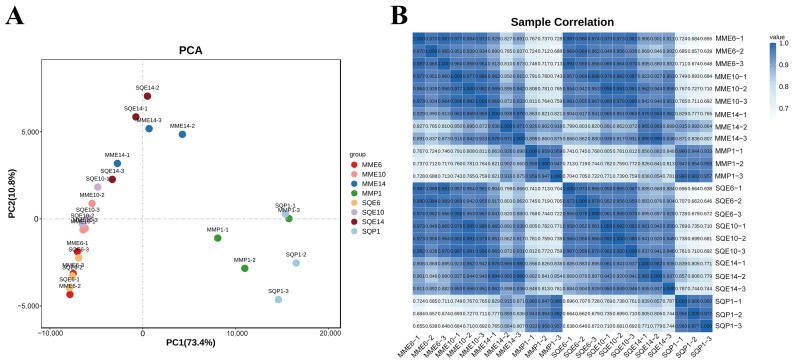
Global gene expression patterns of SQ and MM during E6-P1. (**A**) PCA analysis of all the genes in SQ and MM during E6-P1; (**B**) correlation analysis of all gene expression patterns in SQ and MM during E6-P1.

After quality control of the sequencing data from the 24 samples, all of them had more than 97.28% of Q20 and more than 92.67% of Q30. Comparing the validated data with the reference genome, the ratio of clean reads ranged from 75.79% to 81.49% (Supplementary Table S3). Based on the FPKM value of each gene (Supplementary Table S7), the expression distribution of genes or transcripts from different samples was shown by the expression distribution map. The gene expression abundance map (Supplementary Figure S1) and violin map (Supplementary Figure S2) indicate the accuracy of subsequent analysis. The PCA results showed that the two breeds differed greatly in gene expression trends between all embryonal days and P1 stages, while the gene expression trends on the E6 and E10 stages were less different (Figure 2A), and the clustering diagram showed a high correlation between the samples of both pigeon breeds in the same stage (Figure 2B), which ensured the accuracy of the subsequent analysis.

### 3.3. Analysis of DEGs

**Figure 3:**
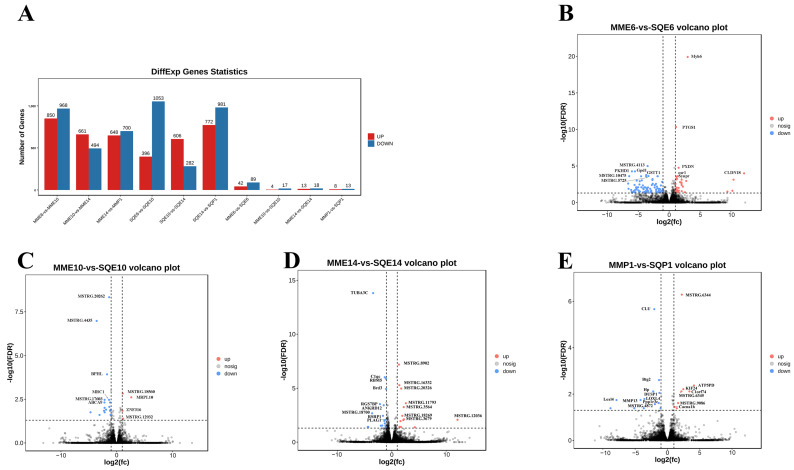
Detailed number of DEGs and volcano plots of DEGs between pigeon breeds at the same stage. (**A**) Detailed number of DEGs in comparison between breeds at the same stage or between stages in the same breed; (**B**) volcano map of DEGs from MME6-VS-SQE6; (**C**) volcano map of DEGs from MME10-VS-SQE10; (**D**) volcano map of DEGs from MME14-VS-SQE14; (**E**) volcano map of DEGs from MMP1-VS-SQP1.

**Figure 4:**
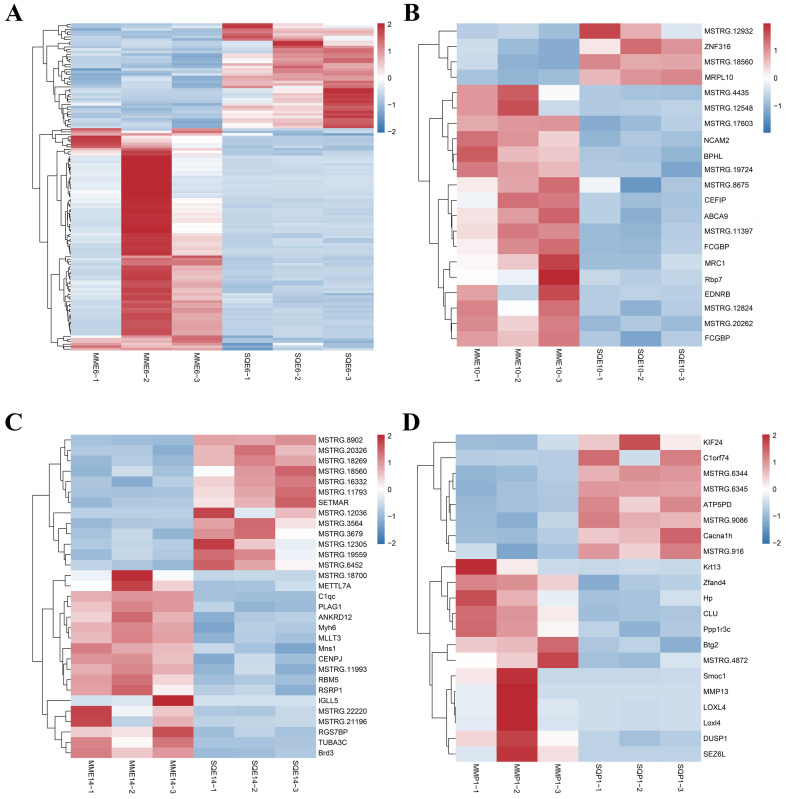
Heatmap of DEGs in comparison between pigeon breeds at the same stage. (**A**) heatmap of DEGs from MME6-VS-SQE6; (**B**) heatmap of DEGs from MME10-VS-SQE10; (**C**) heatmap of DEGs from MME14-VS-SQE14; (**D**) heatmap of DEGs from MMP1-VS-SQP1.

Based on the results of differential analysis, DEGs were screened, and the criterion for differential expressed gene screening was FDR < 0.05, |log2FC| > 1. The differential expression analysis was performed on pectoral muscle from three pigeons per breed per stage (*n* = 3). A total of 204 DEGs were obtained in comparison between breeds at the same stage, among which 42 up-regulated genes and 89 downregulated genes were found in MME6-VS-SQE6, and the most significant DEGs were *MYH6* and *PTGS1*. There were 4 up-regulated genes and 17 downregulated genes in MME10-VS-SQE10, and the most significant DEGs were *BPHL* and *MRPL10*. There were 13 up-regulated and 18 downregulated genes in MME14-VS-SQE14, among which *UTBA3C* and *C1QC* were the most significant DEGs. MMP1-VS-SQP1 had 8 up-regulated genes and 13 downregulated genes, and the most significant DEGs were *CLU* and *BTG2* (Figure 3A–E, Supplementary Table S8). Clustered heatmap analysis was performed for genes with significantly up-regulated and significantly downregulated expression in each comparison group. The heatmaps showed that each replicate in the same group was clustered together, indicating the reliability of sample collection (Figure 4A–D).

### 3.4. GO and KEGG Enrichment Analysis

**Figure 5:**
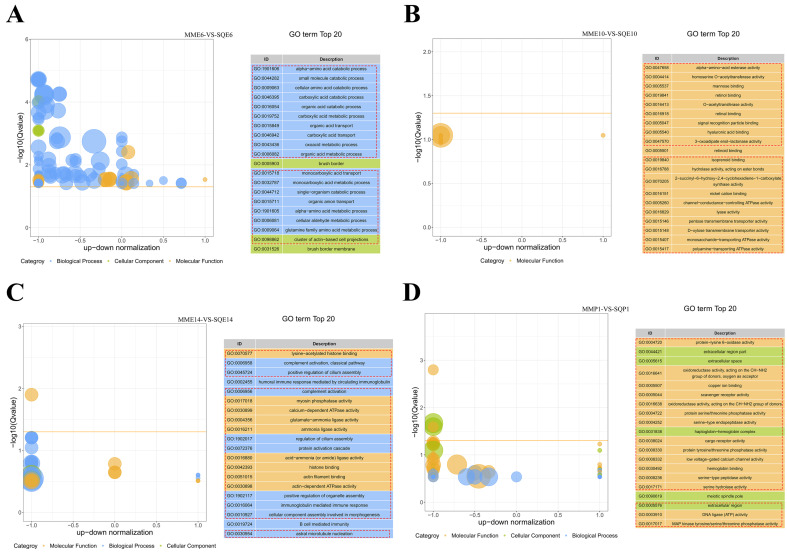
GO enrichment analysis of DEGs in comparison between pigeon breeds at the same stage. (**A**) Top 20 GO enrichment analysis of DEGs from MME6-VS-SQE6; (**B**) top 20 GO enrichment analysis of DEGs from MME10-VS-SQE10; (**C**) top 20 GO enrichment analysis of DEGs from MME14-VS-SQE14; (**D**) top 20 GO enrichment analysis of DEGs from MMP1-VS-SQP1.

To explore the function of the DEGs, we performed GO and KEGG analyses of the up- and downregulated DEGs in each comparison group. The top 20 GO enrichment items in MME6-VS-SQE6 related to muscle development were GO:0009063 cellular amino acid catabolic process, GO:0046395 carboxylic acid catabolic process and GO:0016054 organic acid catabolic process (Figure 5A). The top 20 GO enrichment items in MME10-VS-SQE10 related to muscle development were GO:0047658 alpha-amino-acid esterase activity, GO:0005540 hyaluronic acid binding and GO:0047570 3-oxadipate enol-lactonase activity (Figure 5B). The top 20 GO enrichment items in MME14-VS-SQE14 related to muscle development were GO:0070577 lysine-acetylated histone binding, GO:0002455 humoral immune response mediated by circulating immunoglobulin mediated and GO:0017018 myosin phosphatase activity (Figure 5C). There were no muscle development-related top 20 GO enrichment items in MMP1-VS-SQP1 (Figure 5D).

The pathway-based analysis is helpful to further understand the biological functions of DEGs. Pathways enriched in MME6-VS-SQE6 associated with muscle development were ko00350: Tyrosine metabolism and ko00380: Tryptophan metabolism. Pathways enriched in MME10-VS-SE10 associated with muscle development were ko04514: cell adhesion molecules and ko04020: calcium signaling pathway. The pathways associated with muscle development enrichment in MME14-VS-SQE14 were ko04260: cardiac muscle contraction and ko04261: adrenergic signaling in cardiomyocytes. The enriched pathways related to muscle development in MMP1-VS-SQP1 included ko04020: calcium signaling pathway; ko04010: MAPK signaling pathway; ko04260: cardiac muscle contraction; ko00190: oxidative phosphorylation (Supplementary Table S9). Their KEGG enrichment circle maps are shown in Supplementary Figure S3.

### 3.5. Construction of Gene Interaction Network

**Figure 6:**
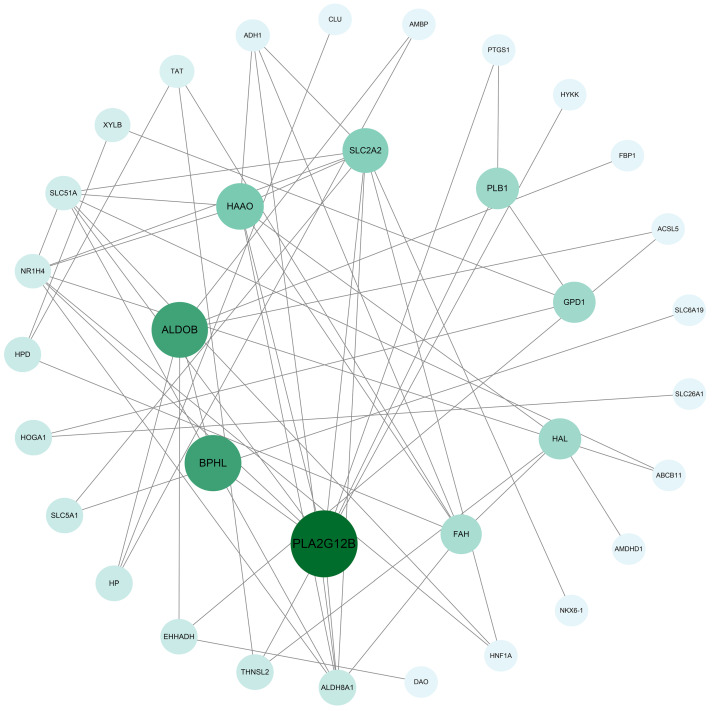
STRING-based gene interaction network construction. Predicted interaction network of myogenic DEGs. The larger the circle and the darker the color, the greater the genetic association.

To further explore the interactions between genes that differ in the mechanisms of skeletal muscle development between two pigeon breeds, the DEGs involved in muscle development-related biological processes by GO and KEGG analysis were utilized to predict the gene interaction network using the *Columba livia* database in STRING website and visualized using Cytoscape software. 33 Hub genes were screened based on the interaction network graph, including phospholipase a2 group xiib (*PLA2G12B*), biphenyl hydrolase like (*BPHL*), aldolase, fructose-bisphosphate b (*ALDOB*), 3-hydroxyanthranilate 3, 4-dioxygenase (*HAAO*) and solute carrier family 2 member 2 (*SLC2A2*) (Figure 6).

### 3.6. Validation of Myogenic DEGs by RT-qPCR

**Figure 7:**
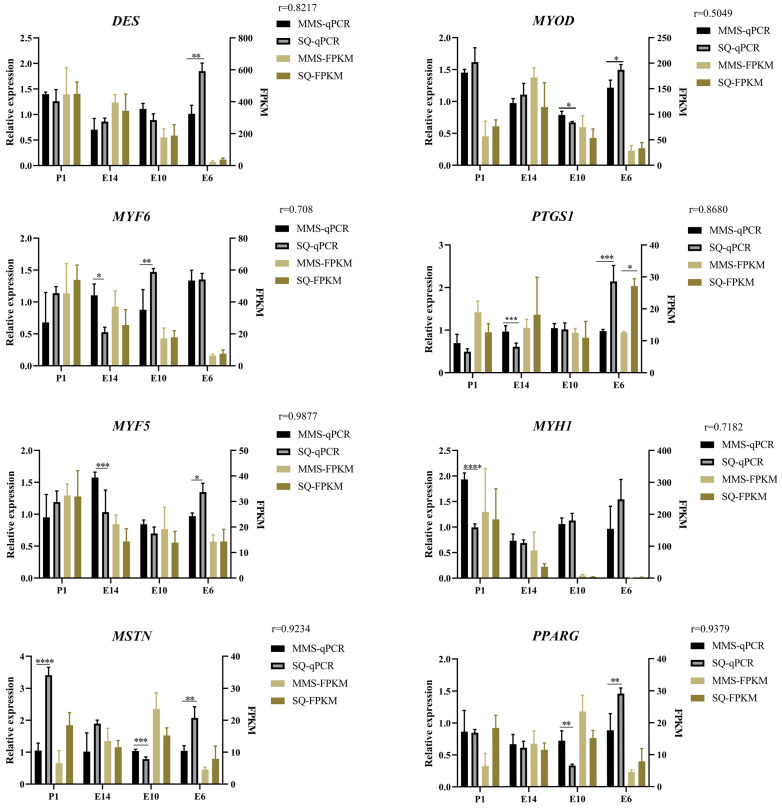
Validation of myogenic DEGs using RT-qPCR. RT-qPCR of *DES*, *MYOD*, *MYF6*, *PTGS1*, *MYF5*, *MYH1*, *MSTN* and *PPARG* in SQ and MM during E6-P1. *, *p* < 0.05; **, *p* < 0.01; ***, *p* < 0.001; ****, *p* < 0.0001. The closer the absolute value of r is to 1, the stronger the linear correlation between the two variables.

In order to verify the gene expression levels in the skeletal muscle of two pigeon breeds, we selected eight myogenic genes, *DES*, *MYOD*, *MYF6*, *PTGS1*, *MYF5*, *MYH1*, *MSTN* and *PPARG*, which are important for embryonic muscle development or showed significantly differential expression patterns between two pigeon breeds, and quantified their expression levels by RT-qPCR. The melting curves of these genes are shown in Supplementary Figure S4. The relative expression of *DES* on E6 was significantly higher in the Shiqi pigeon than in the European meat pigeon. The expression of *MYOD* was lower in European meat pigeons, except on E10 when it was higher than in Shiqi pigeons. *MYF6* expression was significantly higher in European meat pigeons than in Shiqi pigeons on E14, and the opposite trend was found on E10. *PTGS1* expression was significantly lower in European pigeons than in Shiqi pigeons on E6, and greater in European meat pigeons than in Shiqi pigeons during the other stages. The relative expression level of *MYF5* was significantly higher in European meat pigeons than in Shiqi pigeons on E14, and significantly higher in Shiqi pigeons during E6. The expression of *MYH1* was higher in European meat pigeons than that in Shiqi on P1 and E14, and the relative expression of *MYH1* in European meat pigeons was significantly higher than that in Shiqi pigeons on P1. The relative expression level of *MSTN* in Shiqi pigeons was significantly higher than that in European meat pigeons on P1 and E6, while the opposite was observed in E10. The relative expression level of *PPARG* in the Shiqi pigeon was significantly higher than that in the European meat pigeon on E6, but the relative expression level of this gene in the European meat pigeon was higher than that in the Shiqi pigeon during other stages and reached significance on E10 (Figure 7). The results showed that the expression of these genes had similar down- or up-regulation trends with the results of RNA-seq (correlation coefficient (r) ≥ 0.5049).

## 4. Discussion

The HE staining results showed that the myofiber density and total myofiber number were greater in the Shiqi pigeon than those in the European meat pigeon, and Shiqi pigeons have a greater proportion of smaller diameter myofiber than European meat pigeons. These results suggested that the meat of the Shiqi pigeon may be more tender than that of the European meat pigeon. Previously, it was shown that at 23 days of age, myofiber diameters were smaller in the male Shiqi pigeon than those in male European meat pigeons, and larger in the female Shiqi pigeon than those in female European meat pigeons, while the situation in myofiber densities was the opposite, but none of them reached significance [26]. The development of myofibers during the embryonic stages may influence the postnatal developmental differences between the two pigeon breeds. The diameter of the myofibers of the Shiqi pigeon was thinner than that of the European meat pigeon both during the embryonic and postnatal stages (male), while the density of the myofibers differed between the male and female of the two pigeon breeds postnatally, but the differences in the density of the myofibers during the embryonic period between the male and female are not yet known as the study did not distinguish gender.

Embryonic muscle development has also been studied in other pigeon breeds and domestic fowls. During the embryonic development of the White King pigeon, the diameter of the pectoral muscle myofiber increased gradually, and the density of the myofiber showed a tendency to increase first and then decrease [27]. HuangYu broiler showed smaller myofiber diameters than the JingNing broiler before the Hamburger and Hamilton stage (HH) 31, and the JingNing chicken showed smaller myofiber diameters compared to both broilers from HH31 until embryonic day 18 [28]. In our previous study, during E15, E23 and P1, the density of myofiber in Shitou geese was significantly greater than that in Wuzong geese, suggesting that Shitou goose muscle contained more myofiber [16].

RNA-seq is an important tool to explore the mechanisms of skeletal muscle development and growth processes in poultry. Chen et al. discovered the secretion by cell, regulation of secretion by cell, regulation of secretion by cell and other pathways in the rapid growth group and the slow growth group of Jinghai yellow chickens, while the herpes simplex infection, p53 signaling pathway and other pathways have been found in KEGG [29]. HU et al. found that oxidative phosphorylation, ecm receptor interaction, focal adhesion, carbon metabolism and amino acid biosynthesis were involved in the regulation of skeletal muscle development in Peking ducks [11]. Our previous study found that the enrichment pathways of Shitou geese and Wuzong geese at different embryonic developmental stages were striated muscle contraction, positive regulation of myotube differentiation, and myoblast proliferation [16]. Some of the pathways found in geese were common in this study, which may be due to similarities in muscle development among birds.

Gene interaction network showed that *CLU*, *ABCB11*, *PTGS1*, *NR1H4*, *HNF1A*, *NKX6-1* and other genes were involved in muscle development processes, among which some genes have been explored. Zhang et al. found that *CLU* played a prominent role in regulating several fundamental physiological processes, including programmed cell death, metastasis, invasion, proliferation and cell growth [30]. Prostaglandins (PGS) may regulate muscle regeneration by influencing inflammation processes and be involved in various stages of muscle formation in vitro [31]. Yang et al. found that nuclear receptor subfamily 1, Group H, member 4 (*NR1H4*) is a bile acid receptor that plays an important role in regulating energy metabolism in human and animal liver, muscle and adipose tissue [32]. Pig *HNF1A* gene polymorphism can be used as a candidate marker to improve meat quality and carcass quality traits [33]. There are also some less-reported genes in muscle development, such as *PTGS1* and *NXK6-1*. These genes are less studied in pigeon muscle development and can be studied at the cellular level to complement the gene regulatory network of pigeon muscle development.

We selected eight genes, *DES*, *MYOD*, *MYF6*, *PTGS1*, *MYF5*, *MYH1*, *MSTN* and *PPARG* as RNA-seq data validation, and the results were consistent with the overall trend of RNA-seq, and the correlation coefficient was high. *DES* contributes to muscle structure and cellular integrity [34], on E6 it showed a significantly higher expression level in the Shiqi pigeon than that in the European meat pigeon by RT-qPCR method and during E10-P1, the *DES* expression of the two pigeon breeds was high under the two methods but showed no significant differences. *MYOD* was expressed in greater amounts in the Shiqi pigeon than in the European meat pigeon on E6, and the reverse situation was found on E10, and *MYOD* was the first tissue-specific factor found to be capable of converting non-muscle somatic cells into skeletal muscle cells [35]. The expression of *DES* and *MYOD* was higher on E6 in the Shiqi pigeon than in the European meat pigeon, whereas the opposite was found on E10, which may indicate that muscle development is faster in the Shiqi pigeon than in the European meat pigeon on E10. *MYF6* established ligand/receptor interactions between muscle stem cells and their associated myofiber [36]. *MYF6* expression was higher in both pigeon breeds on P1, and the relative expression of this gene was lower in the Shiqi pigeon than that in the European meat pigeon only on E14, which may indicate that the muscle development of Shiqi pigeon is slower than that of European meat pigeon on E14. The endogenous production of prostaglandins (PG) by skeletal muscle myoblasts played an important role in muscle growth and differentiation and also cooperated with *PTGS1* in the synthesis of prostaglandins [37]. The expression of *PTGS1* tended to decrease with the embryonic development of both pigeon breeds and was higher on E6 in the Shiqi pigeon than in the European meat pigeon, which suggests that the role of *PTGS1* may have led to a significantly higher myofiber density in the Shiqi pigeon than in European meat pigeon on E6. *MYF5* is recognized as a myogenic determinant that directs progenitor cells to establish skeletal muscle lineages [38]. The expression of *MYF5* in European meat pigeon was greater than that in Shiqi pigeon on E10 and E14, which may indicate that European meat pigeons develop muscle faster than Shiqi pigeons in the middle and late stages of embryonic muscle development. Myosin heavy chain (*MYHC*) isoforms affect the contraction relaxation activity of skeletal muscles and are involved in determining muscle composition; among them, *MYH1* is related to skeletal muscle contraction [39]. On E14 and P1, the expression level of *MYH1* in European meat pigeon was higher than that in Shiqi pigeon, which may indicate that in the later stages of embryonic development, the myofiber differentiation speed of European meat pigeons was faster than that of Shiqi pigeons. Myostatin (*MSTN*), a negative regulator of skeletal muscle growth and development that inhibits myoblast proliferation, is an important candidate gene for animal breed improvement [40]. The expression of *MSTN* in the Shiqi pigeon was greater than that of the European meat pigeon on E6 and P1, which suggests that the muscle development of the Shiqi pigeon may be slower than that of the European meat pigeon at the initial and final stages of embryonic muscle development. Peroxisome proliferator-activated receptors (*PPAR*) are a group of transcription factors associated with cellular functions, including cell proliferation and differentiation [41], and *PPARG* is essential for muscle stem cell function and muscle repair [42]. The expression of *PPARG* on E6 is higher in Shiqi pigeons than that in European meat pigeons, similar to the previous genes, suggesting that the early muscle development of Shiqi pigeon may be faster than that of European meat pigeon. However, the functions of these candidate genes need to be further studied in the future.

## 5. Conclusions

In this study, we analyzed the differences in the development of the pectoral muscles of the European meat pigeon and Shiqi pigeon at different developmental stages during the embryonic period. A number of GO and KEGG pathways were identified from the transcriptomic data, gene interaction networks were constructed and some genes were screened for validation. This study laid the foundation for unraveling the distinct mechanisms of pectoral muscle development during the embryonic stages between two pigeon breeds.

## Data Availability

The raw RNA-seq data have been submitted to the SRA database under PRJNA1005959 (http://www.ncbi.nlm.nih.gov/Traces/sra/ (accessed on 16 September 2023)).

## Supplementary Materials

The following supporting information can be downloaded at: https://www.mdpi.com/article/10.3390/ani13203267/s1, Figure S1: The gene expression abundance map. Based on the FPKM value of each gene, the expression distribution of each sample gene or transcript is displayed through an expression distribution map. Figure S2: Violin map. Visualization of gene abundance expressed in different samples of two pigeon breeds at different stages. Figure S3: KEGG enrichment cycle diagram. (A) KEGG analysis of DEGs from MME6-VS-SQE6; (B) KEGG analysis of DEGs from MME10-VS-SQE10; (C) KEGG analysis of DEGs from MME14-VS-SQE14; (D) KEGG analysis of DEGs from MMP1-VS-SQP1. The first circle: enriches the top 20 pathways, with the outer circle being the coordinate ruler for the number of DEGs. Different colors represent different A classes. Circle 2: the number and Q value of the pathway in the background of the DEGs. The more the number of differential gene backgrounds, the longer the bars and the smaller the Q value, the redder the color. The third circle: a bar chart of the proportion of up-regulated DEGs, with dark purple representing the proportion of up-regulated DEGs and light purple representing the proportion of downregulated DEGs; the specific numerical values are displayed below. Circle 4: rich factor values for each path (the number of DEGs in that path divided by all the numbers in that path), background grid lines, each grid representing 0.1. Figure S4: The qPCR melting curves of eight genes for RT-qPCR. Table S1: The histological statistic analysis data of H&E staining. Table S2: Interaction strength between genes in comparison between two breeds of pigeons. Table S3. Reads mapping data of all the samples in RNA-Seq sequencing data. Table S4: Number of reads involved in the quantification of each sample. Table S5: Interaction strength between genes in comparison between two pigeon breeds. Table S6: The RT q-PCR primers used in this study. Table S7: Gene expression at different times in two pigeon breeds presented as raw reads count and FPKM. Table S8: Differential expression results for each comparison groups. Table S9: Summary of KEGG related to muscle development in 4 stages. Pathways associated with muscle development are highlighted in red.
