# Supplementary figures and images for: Transcriptomic Analysis on Pectoral Muscle of European Meat Pigeons and Shiqi Pigeons during Embryonic Development
Source: Animals (Basel). 2023 Oct 19;13(20):3267. doi: 10.3390/ani13203267 (PMC10603743; doi:10.3390/ani13203267)

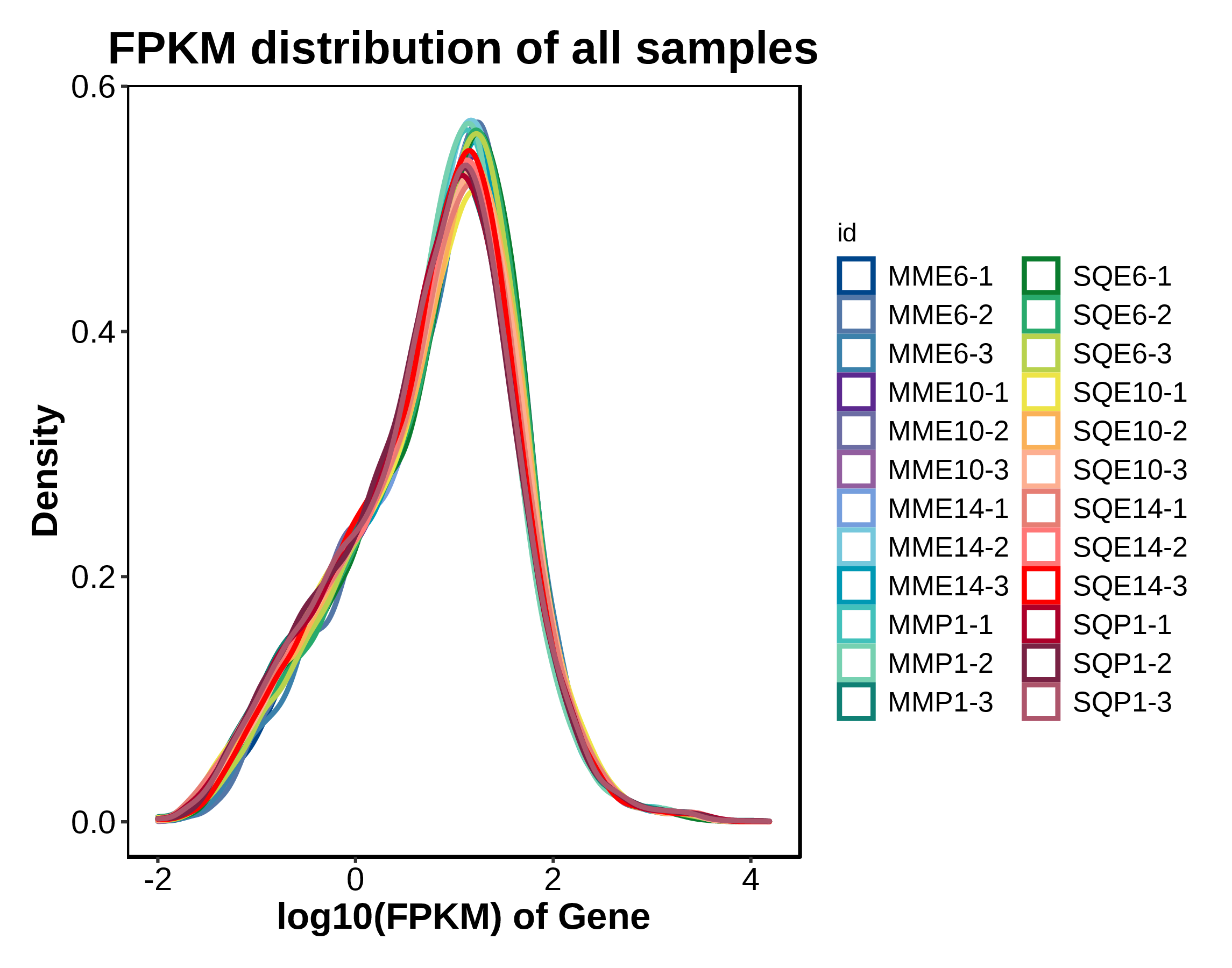

Supplement: Supplementary file 1 [file animals-13-03267-s001.zip › Figure S1.tif]

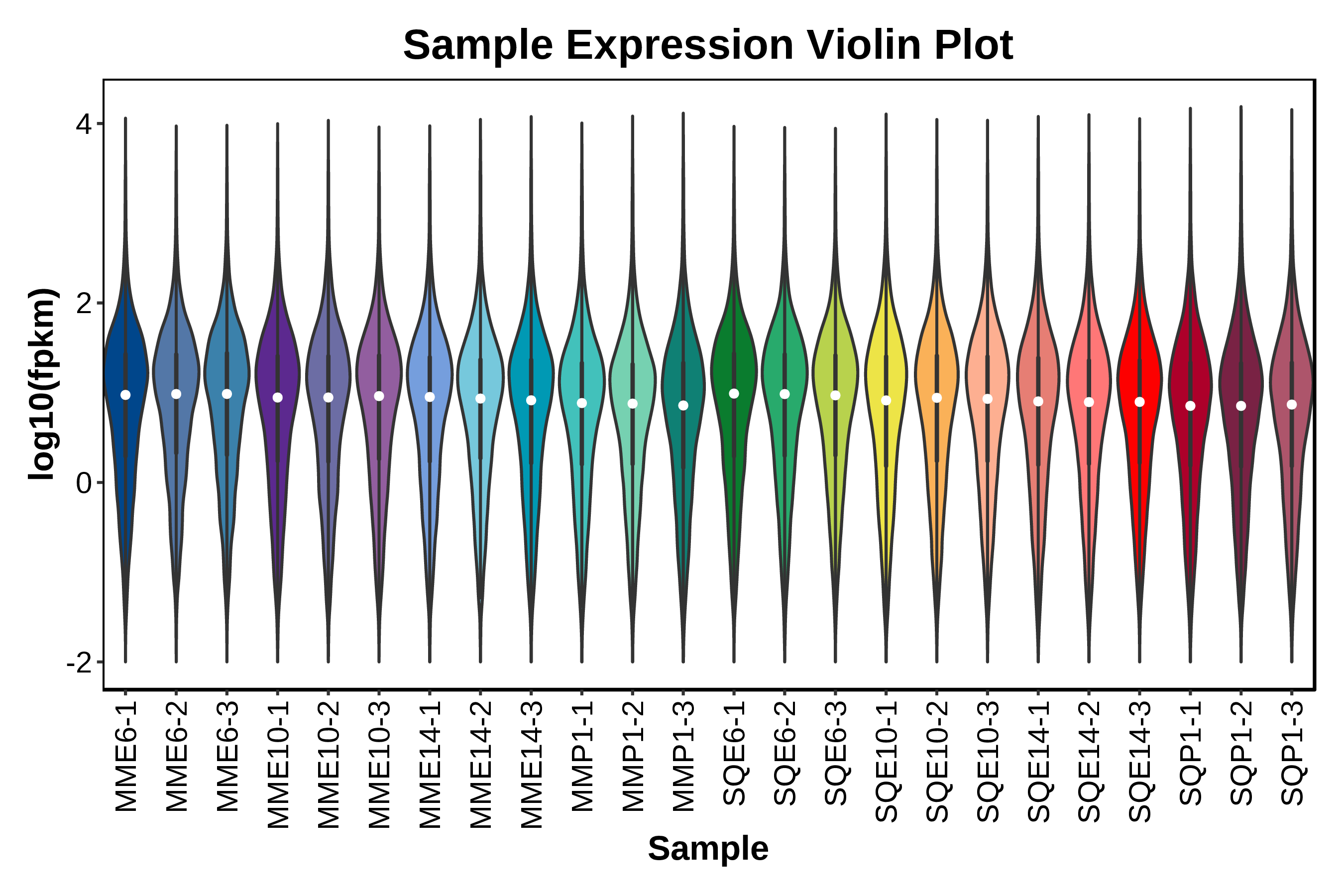

Supplement: Supplementary file 1 [file animals-13-03267-s001.zip › Figure S2.tif]

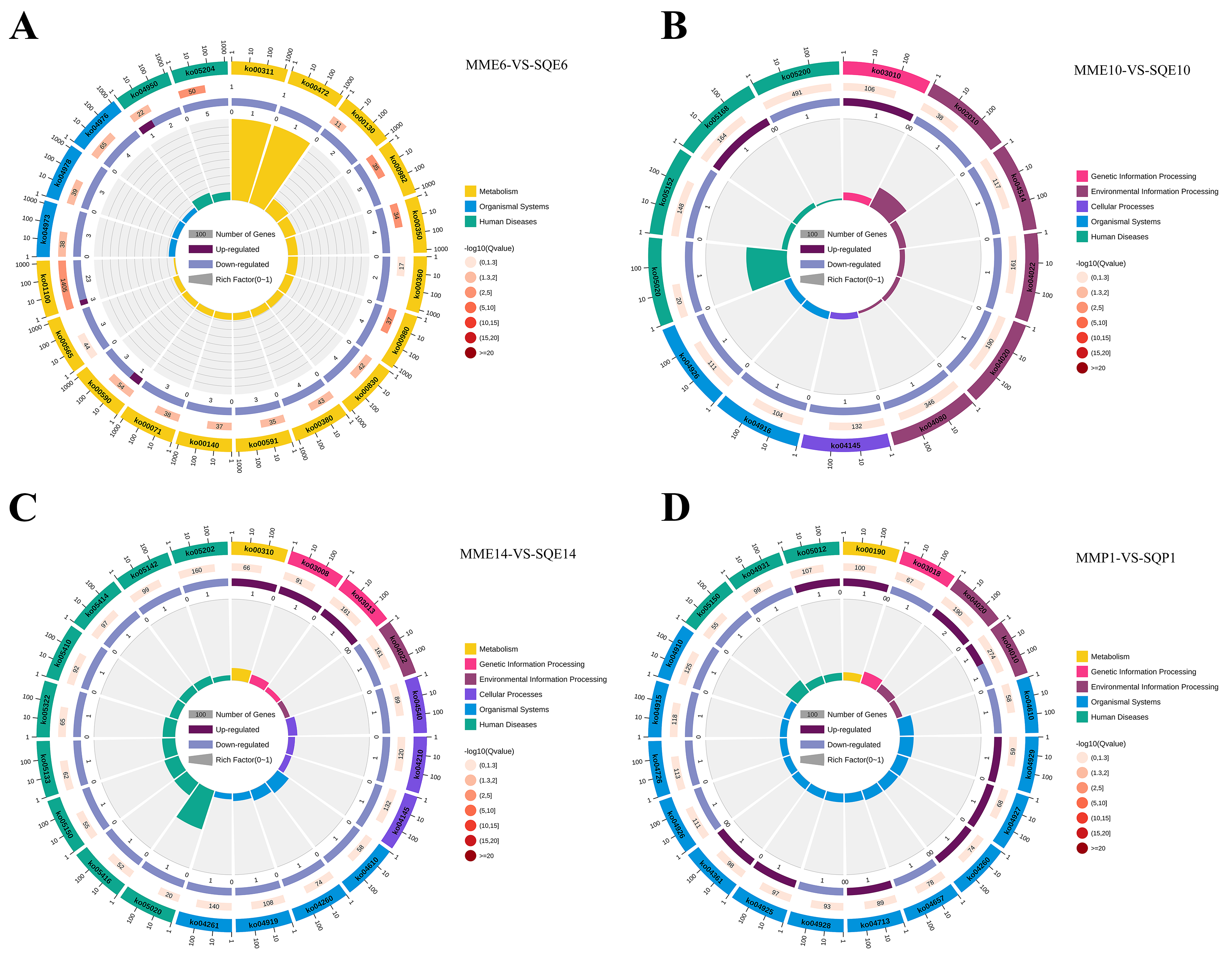

Supplement: Supplementary file 1 [file animals-13-03267-s001.zip › Figure S3.tif]

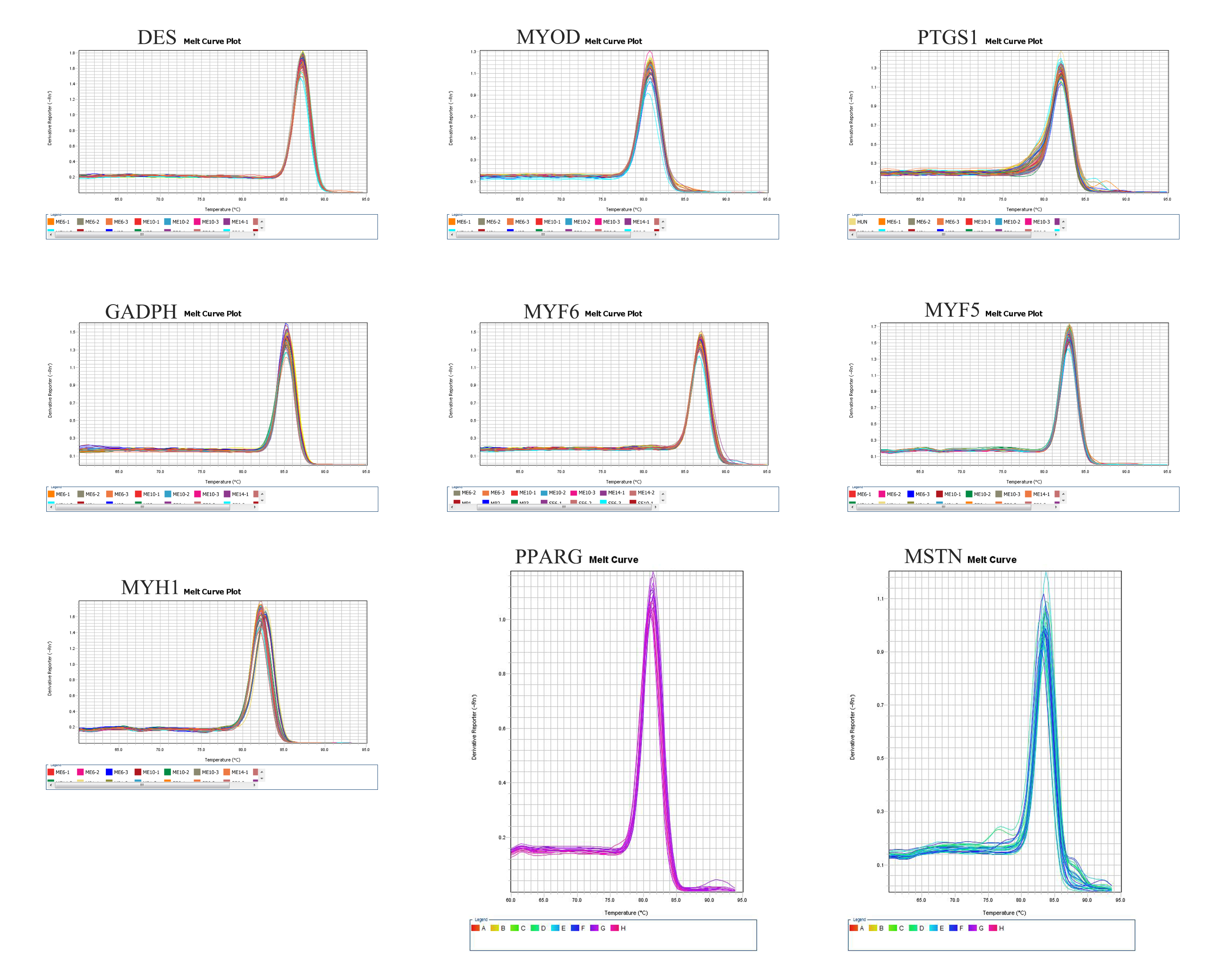

Supplement: Supplementary file 1 [file animals-13-03267-s001.zip › Figure S4.tif]
